# Supplementary material for: LICHEN enables light-chain immunoglobulin sequence generation conditioned on the heavy chain and experimental needs
Source: Commun Biol. 2026 Feb 21;9:468. doi: 10.1038/s42003-026-09727-3 (PMC13036001; doi:10.1038/s42003-026-09727-3)
Supplement: Supplementary file 1 — Supplementary Information [file 42003_2026_9727_MOESM1_ESM.pdf]

Supplementary Information

LICHEN enables Light-chain Immunoglobulin sequence generation Conditioned on the Heavy chain and Experimental Needs

Henriette L. Capel[1], Isaac Ellmen[1], Chris J. Murray[2], Giulia Mignone[2], Megan Black[2], Brendan Clarke[2], Conor Breen[2], Sean Tierney[2], Patrick Dougan[2], Richard J. Buick[2], Alexander Greenshields-Watson[1], and Charlotte M. Deane[1,✉]

[1]Oxford Protein Informatics Group, Department of Statistics, University of Oxford, 24-29 St Giles', Oxford, OX1 3LB United Kingdom
[2]Fusion Antibodies plc, Springbank Industrial Estate, 1 Springbank Road, Dunmurry, Belfast, BT17 0QL United Kingdom

Name	Numbering issues (ANARCI)	Numbering issues (ANARCII)	Non human (Hu-mAb)	Non human (Humatch)	CDR length outside natural distribution	Modelling issues (ABB2)
Native	0	0	0	0	0	1
LICHEN	0	0	0	3	0	1
LICHEN (20 repeats)	5	0	8	27	0	-

Table S1. LICHEN generates valid (antibody-like) light sequences. For all entries in the standard_ds one ("LICHEN") and 20 ("LICHEN (20 repeats)") generated light sequences by LICHEN were validated based on the ability of ANARCI[1] and ANARCII[2] to number the sequence, humanness according to Hu-mAb[3] and Humatch[4], the number of CDRs outside a natural distribution and the ability of ABB2[5] to structurally model the sequence. Results are compared against the native light sequences.

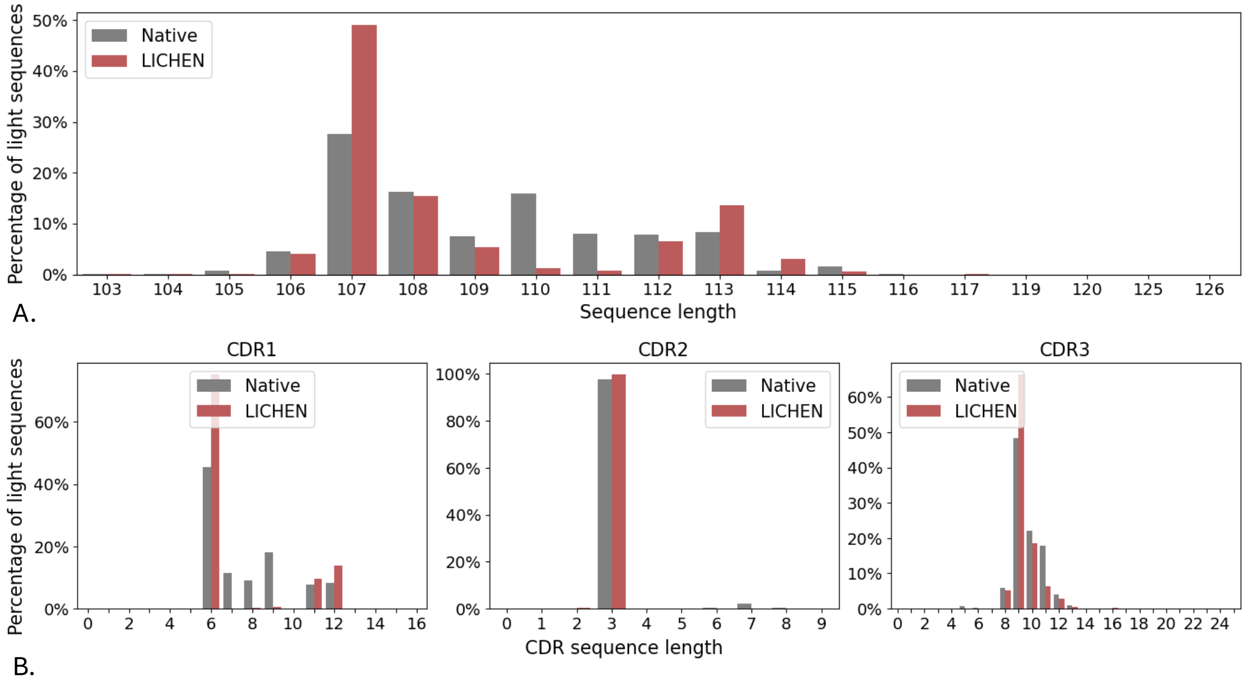

Figure S1. The full VL (A) and light CDR (B) sequence length of all the generated light sequences by LICHEN (red) compared to the native light sequences (grey) in the standard_ds. The percentage of light sequence with a certain length are shown.

Name	Numbering issues (ANARCI)	Numbering issues (ANARCII)	Non human (Hu-mAb)	Non human (Humatch)	CDR length outside natural distribution
Native	0	0	0	0	0
LICHEN	0	0	2	5	0
p-IgGen	9	2	31	41	0

Table S2. Validity of generated light sequences by LICHEN and p-IgGen[10] compared to the native light sequences in the comparison_ds. Sequences are validated based on the ability of ANARCI[1] and ANARCII[2] to number the sequence, humanness according to Hu-mAb[3] and Humatch[4], and the number of CDRs outside a natural distribution.

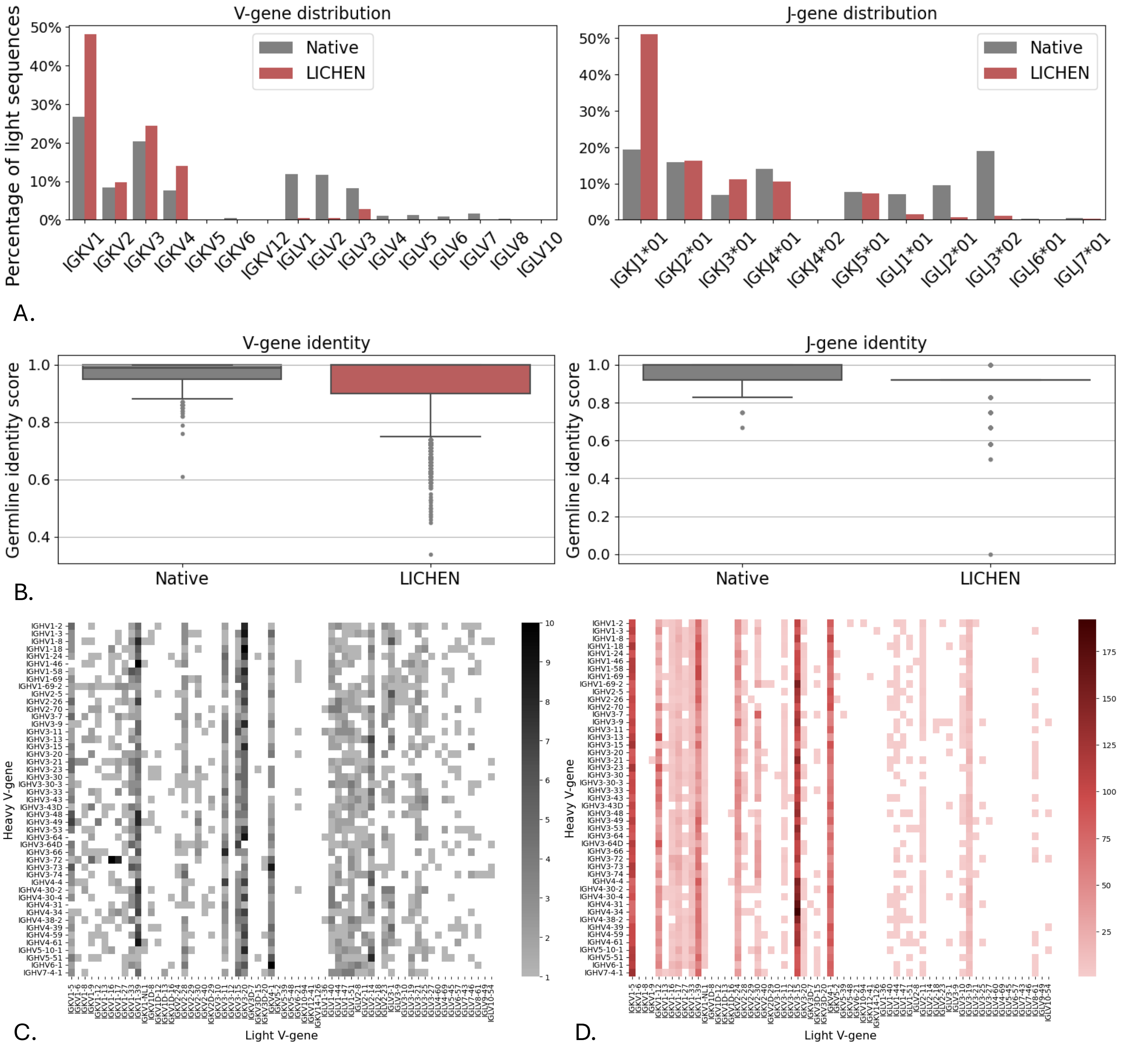

Figure S2. The germline diversity of sequences generated by LICHEN. (A) The V-gene family and J-gene diversity of the generated light sequences for the standard_ds (red) are compared against the native sequences (grey). (B) The germline identity score of these generated sequences (red) are also compared to the native sequences (grey). (C) The native germline pairing diversity of 50 samples of each of the 47 heavy V-genes observed in the test set (the genes_ds). (D) The germline pairing of the ten generated sequences per heavy sequences in the genes_ds. The closest V-gene and J-gene and the identity scores were determined by ANARCI[1].

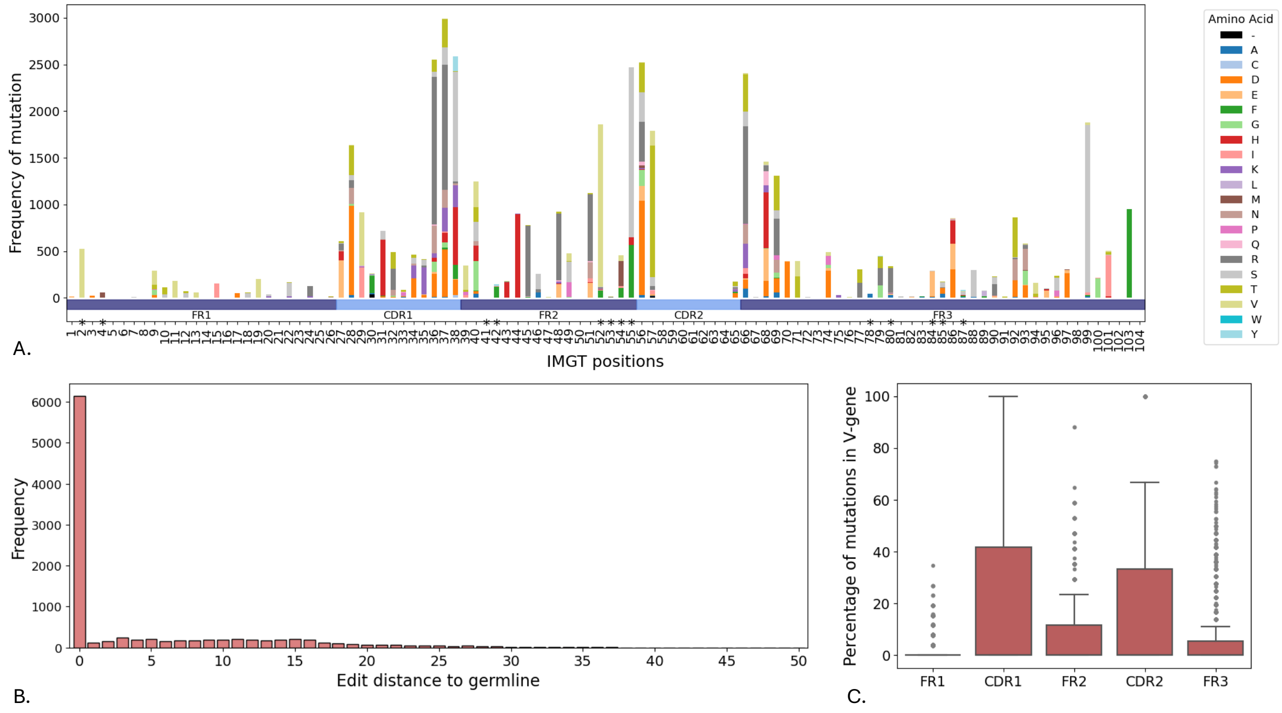

Figure S3. Mutations in the generated sequences from the closest V-gene germline in the standard_ds. For every 500 heavy sequences 20 light sequences were generated resulting in a set of 10,000 generated light sequences. (A) The number of mutations including amino acid frequencies are shown per IMGT position. Amino acids mutations and deletions (indicated by "-") are coloured. The CDRs (light blue) and FRs (dark blue) according to the IMGT definition are highlighted in the rectangle and positions identified as belonging to the Vernier zone are indicated with asterisks on the x-axis. (B) The edit distance to closest germline V-gene are shown for the generated sequences by LICHEN. (C) The percentage of mutations for each generated sequences are shown per region. 100% indicates that all positions belonging to a specific region are mutated in a sequence.

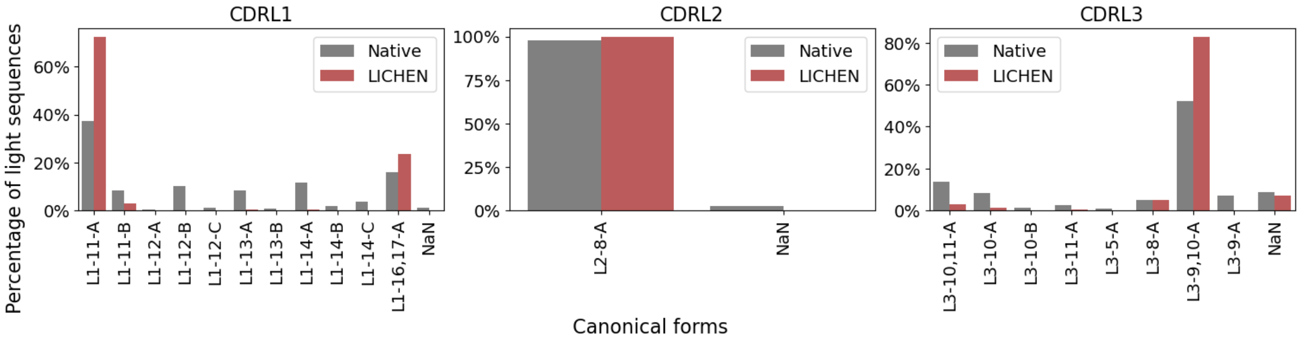

Figure S4. The canonical form assignment by SCALOP[6] on all generated light sequences by LICHEN (red) compared to the native light sequences (grey) in the standard_ds. The percentage of light sequence with a certain length are shown.

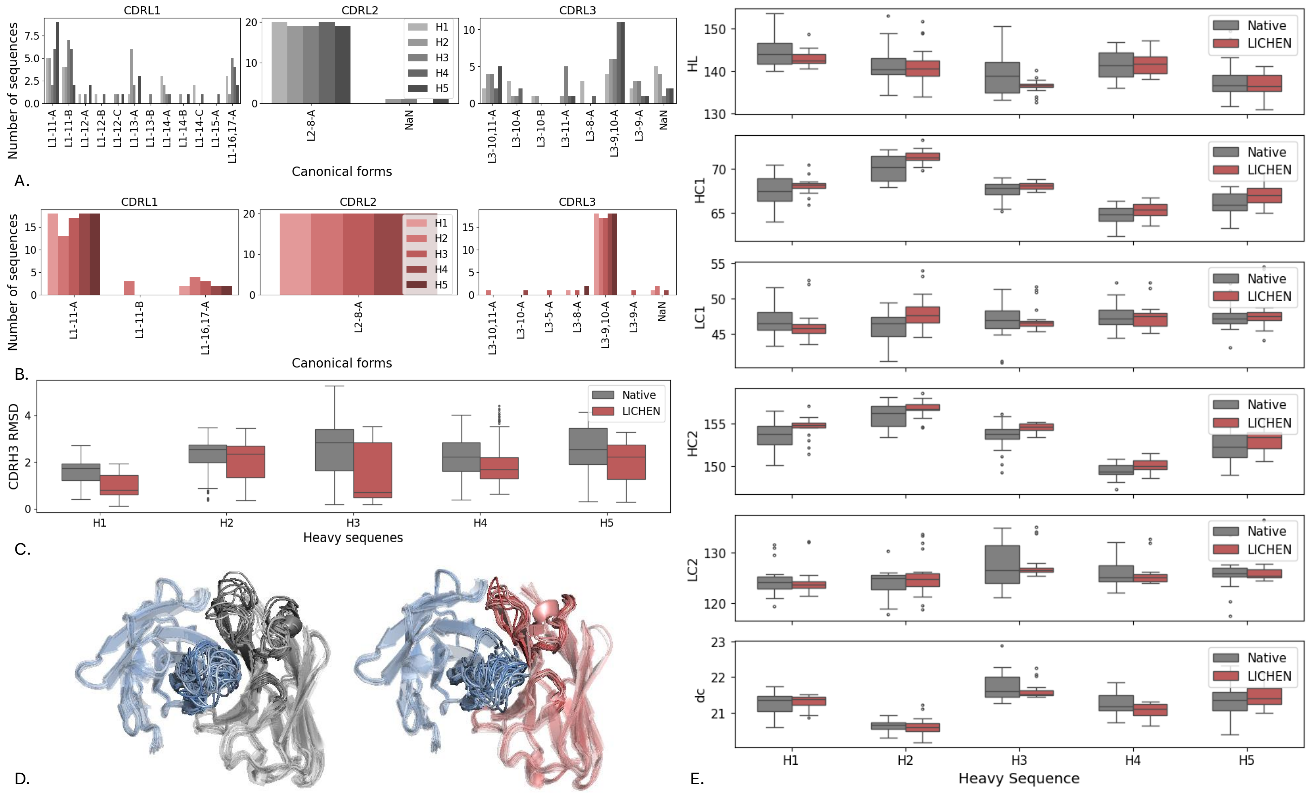

Figure S5. Structural diversity of 20 generated (red) and 20 native (grey) light sequences pairing with 5 different heavy sequences of different V-genes: IGHV3 (H1), IGHV6 (H2), IGHV5 (H3), IGHV1 (H4), and IGHV4 (H5). (A and B) The canonical form assignment of these 20 sequence per heavy chain by SCALOP[6] (C) The RMSD of the unaltered 20 CDRH3 sequences per heavy chain upon light sequence pairing. (D) Structural diversity of light chain CDRs (red) and CDRH3 (blue) of the 20 antibodies of H4 are visualised with Pymol[7]. (E) Variation in VH-VL orientation of the 20 antibodies per heavy chain determined by ABangle[8].

	**Adalimumab**				**Pembrolizumab**			
**Name**	**V-gene**	**J-gene**	**Potential yield (mg/L)**	**Monodispersity (%)**	**V-gene germline**	**J-gene**	**Potential yield (mg/L)**	**Monodispersity (%)**
Therapeutic	IGKV1-27*01	IGKJ1*01	53	>95	IGKV3-11*01	IGKJ4*01	32	>95
Germline-1	IGKV1-39*01	IGKJ1*01	50	>90	IGKV3-20*01	IGKJ1*01	171	>95
Germline-1+	IGKV1-39*01	IGKJ1*01	54	>95	IGKV3-20*01	IGKJ1*01	18	>95
Germline-2	IGKV1D-33*01	IGKJ1*01	22	>90	IGKV3-11*01	IGKJ1*01	75	>95
Germline-2+	IGKV1D-33*01	IGKJ1*01	51	>95	IGKV3-11*01	IGKJ1*01	9	>95
Germline-3	IGKV7-3*01	IGKJ1*01	58	>90	IGKV7-3*01	IGKJ1*01	147	>95
Germline-3+	IGKV7-3*01	IGKJ1*01	40	>95	IGKV7-3*01	IGKJ1*01	27	>95
LICHEN-1	IGKV1-27*01	IGKJ2*01	63	>95	IGKV1-27*01	IGKJ5*01	17	>60
LICHEN-2	IGKV1-27*01	IGKJ5*01	61	>95	IGKV1-17*03	IGKJ4*01	16	>40
LICHEN-3	IGKV1-27*01	IGKJ2*01	48	>95	IGKV3-11*01	IGKJ5*01	7	-
LICHEN-4	IGKV1-16*01	IGKJ2*01	77	>95	IGKV1-39*01	IGKJ2*01	14	-
LICHEN-5	IGKV3-11*01	IGKJ2*01	45	>95	IGKV3-15*01	IGKJ4*01	43	>95
LICHEN-6	IGKV1-13*02	IGKJ2*01	158	>95	IGKV1-9*01	IGKJ2*01	81	>95
LICHEN-7	IGKV2-30*01	IGKJ2*01	12	>95	IGKV1-9*01	IGKJ4*01	57	>95
LICHEN-8	IGKV3-11*01	IGKJ2*01	51	>95	IGKV2-28*01	IGKJ5*01	52	>90
LICHEN-9	IGKV2-24*01	IGKJ2*01	2	-	IGKV1-39*01	IGKJ3*01	90	>85
LICHEN-10	IGKV1D-8*02	IGKJ2*01	110	>95	IGKV2-24*01	IGKJ4*01	4	-
LICHEN-11	IGKV3-15*01	IGKJ2*01	98	>95	IGKV1-16*01	IGKJ5*01	29	>65
LICHEN-12	IGKV1-5*01	IGKJ2*01	56	>95	IGKV3-15*01	IGKJ4*01	36	>85
LICHEN-13	IGKV1-5*01	IGKJ2*01	77	>95	IGKV3-20*01	IGKJ4*01	39	>90
LICHEN-14	IGKV1-8*01	IGKJ3*01	43	>95	IGKV3-15*01	IGKJ1*01	206	>90
LICHEN-15	IGKV1-12*01	IGKJ1*01	20	>90	IGKV1-27*01	IGKJ3*01	125	>95
LICHEN-16	IGKV1-33*01	IGKJ4*01	8	>95	IGKV1-16*01	IGKJ4*01	36	>80
LICHEN-17	IGKV3-20*01	IGKJ3*01	60	>90	IGKV1-13*02	IGKJ1*01	62	>90
LICHEN-18	IGKV2-24*01	IGKJ4*01	9	>90	IGKV1-5*03	IGKJ1*01	26	>55
LICHEN-19	IGKV1-12*01	IGKJ3*01	71	>95	IGKV2-28*01	IGKJ1*01	98	>85
LICHEN-20	IGKV3-15*01	IGKJ1*01	68	>95	IGKV3-15*01	IGKJ3*01	161	>95
LICHEN-21	IGKV1-12*01	IGKJ2*01	49	>90	IGKV1-13*02	IGKJ3*01	61	>95
LICHEN-22	IGKV1-5*01	IGKJ1*01	20	>85	IGKV1-9*01	IGKJ1*01	55	>95
LICHEN-23	IGKV3-11*01	IGKJ4*01	15	>90	IGKV2-24*01	IGKJ4*01	11	>95

Table S3. The V-gene and J-gene (determined by ANARCI[1]), potential yield, and monodispersity of the experimental validated generated sequences by LICHEN and the control sequences. Monodispersity is based on a SEC analysis.

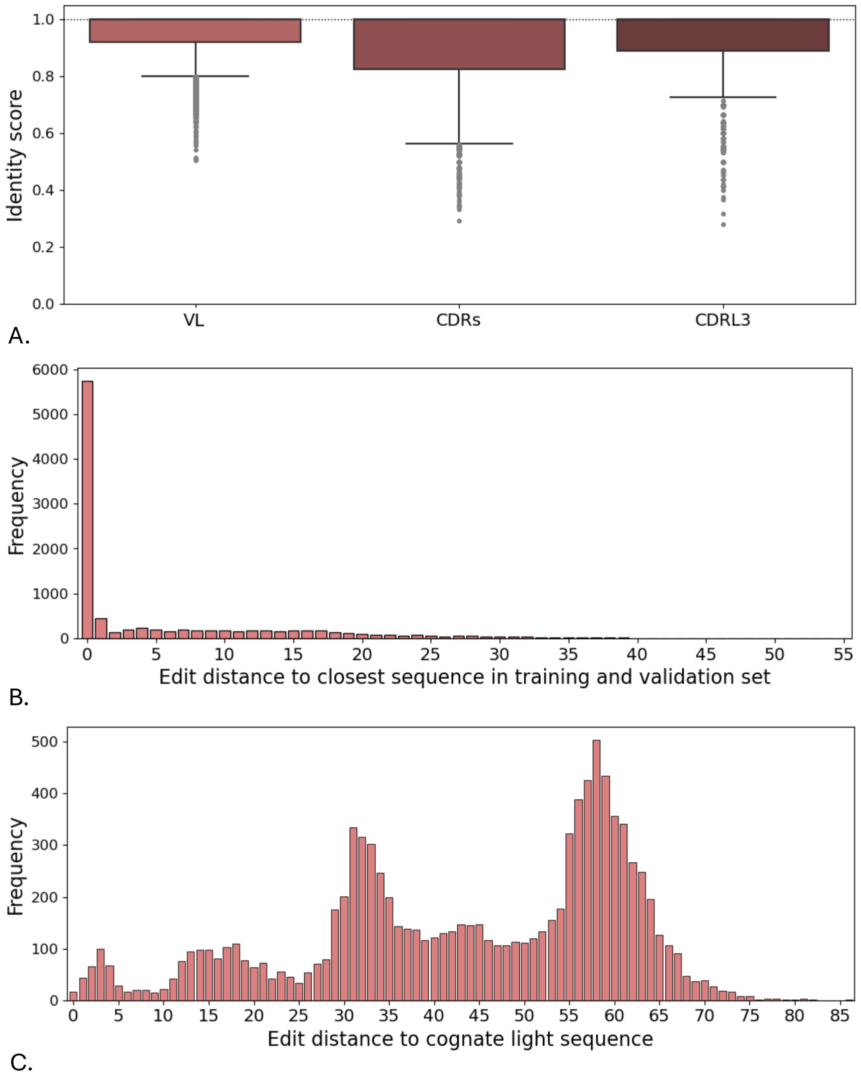

Figure S6. The diversity of generated light sequences compared to sequences observed in the training and validation set. (A) The identity score to the training and validation set of the 20 generated light sequence by LICHEN per heavy chain for the 500 heavy chains in the standard_ds as determined by KAsearch[9]. Identity scores are shown for the full VL, light chain CDRs, and the CDRL3. (B) Edit distance between these 10,000 generated light sequences and observed sequences during training. (C) Edit distances between the 10,000 generated light sequences for heavy sequences observed during training of LICHEN (observed_ds) and the closest cognate light sequence of that heavy chain.

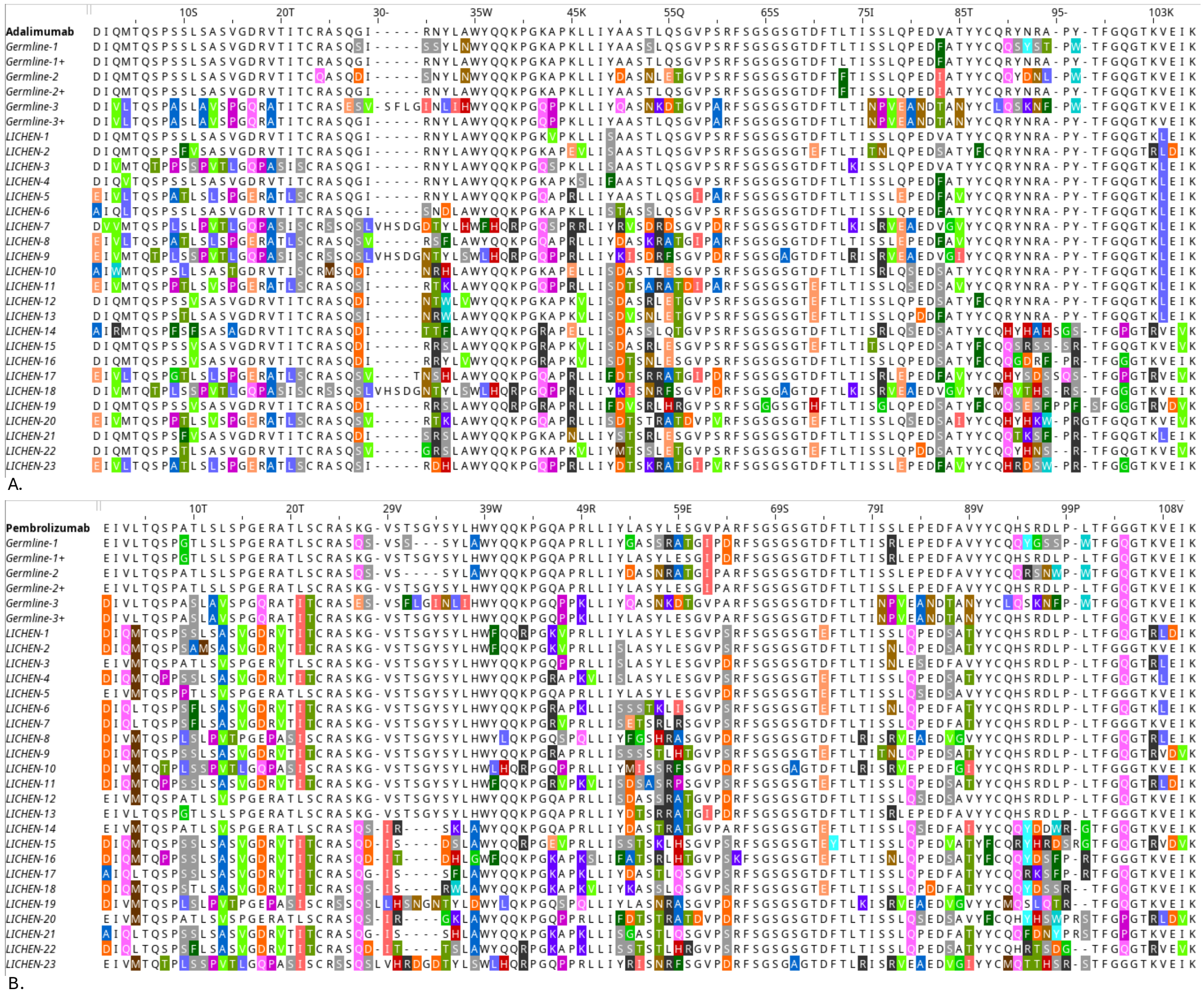

Figure S7. Generated light sequences by LICHEN were experimentally validated on the VH of therapeutics adalimumab (A) and pembrolizumab (B). The therapeutics were used as positive control and germline V-gene sequence combined with IGKJ1*01 (Germline-1/3) as baseline. For adalimumab V-genes IGKV1-39*01, IGKV1D-33*01, and IGKV7-3*01 and for pembrolizumab IGKV3-20*01, IGKV3-11*01, and IGKV7-3*01 were used respectively. The "+" suffix indicates the therapeutic light CDRs (Kabat definition) were grafted into the germline sequences. Three use cases where tested: pairing_CDRs (LICHEN-1 to LICHEN-5), pairing_eCDR (LICHEN-6 to LICHEN-13), and pairing (LICHEN-14 to LICHEN-23). For pairing_CDRs LICHEN was conditioned to all light therapeutic CDRs, for pairing_eCDR LICHEN was conditioned to essential light CDRs only (CDRL3 for adalimumab, CDRL1 and CDRL3 for pembrolizumab). LICHEN was restricted to make only IGKV1, IGKV2, and IGKV3 light sequences. Generated sequences were *in silico* filtered based on ANARCI[1], Humatch[4], Hu-mAb[3], SCALOP[6], ABB2[5], and TAP[11, 12] after which 20 sequences were randomly chosen. Out of those, diverse sequences were selected for experimental validation. Alignments were visualised with Jalview[13] and only differences from the therapeutic light sequence are coloured.

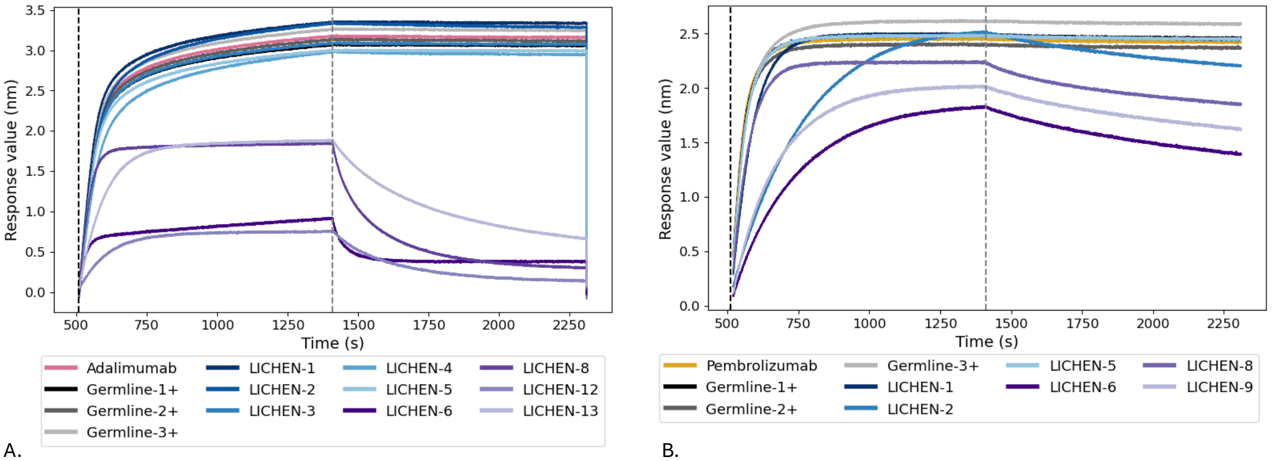

Figure S8. Bio-layer interferometry analysis of the therapeutic antibodies adalimumab (A, pink) and pembrolizumab (B, orange), and the control and generated light sequences pairing with the therapeutic VH sequence showing effective target binding in ELISA. Germline light sequences with grafted CDRs are shown in a grey palette and light sequences generated by LICHEN in a blue palette (all CDRs; pairing_CDRs) and in a purple palette (essential CDRs; pairing_eCDR). The association phase is shown between the dotted black (510 s) and the dotted grey line (1410 s). The dissociation phase is shown after the dotted grey line.

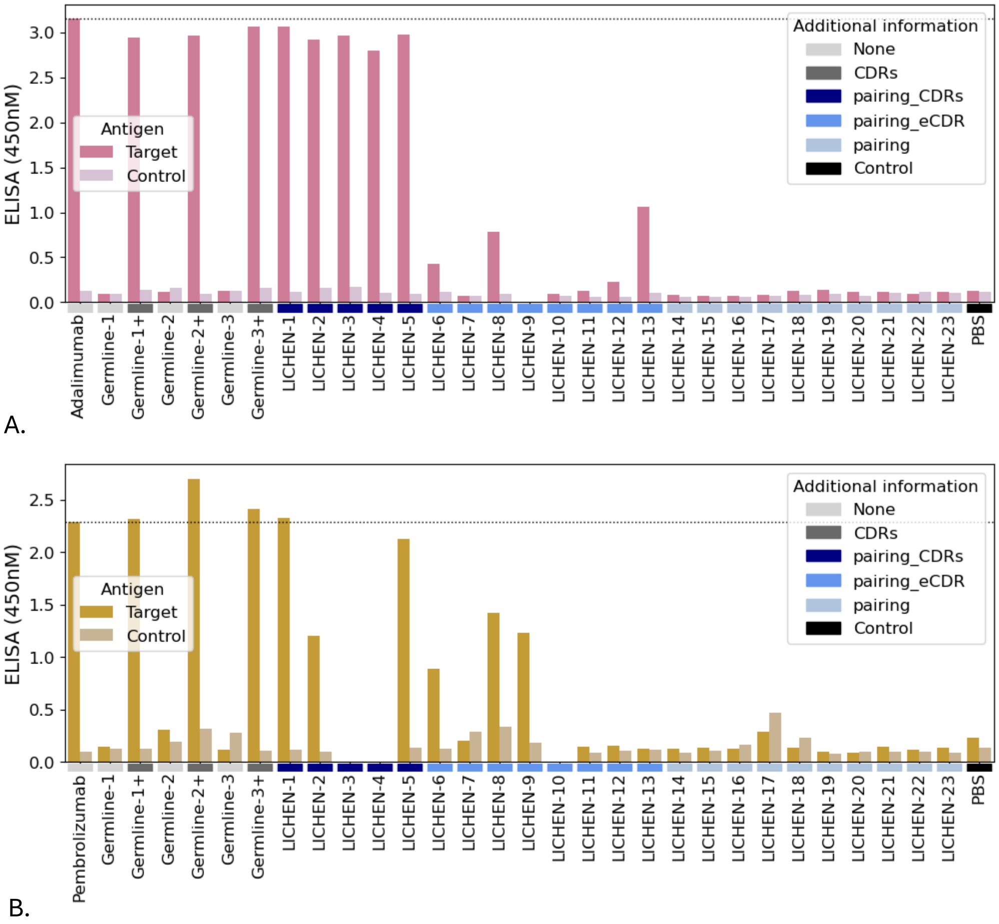

Figure S9. Binding data for the generated light sequences by LICHEN combined with the VH of therapeutics adalimumab (A) and pembrolizumab (B). The therapeutics were used as positive controls. The germline V-gene sequences IGKV1-39*01, IGKV1D-33*01, and IGKV7-3*01 for adalimumab and IGKV3-20*01, IGKV3-11*01, and IGKV7-3*01 for pembrolizumab, labeld "Germline-1", "Germline-2", and "Germline-3" respectively, combined with IGKJ1*01 were used as baselines. All three light CDRs (Kabat definition) were grafted in the germlines labeled with the suffix "+" (dark grey). Three use cases where tested: pairing_CDRs ("LICHEN-1" to "LICHEN-5", navy), pairing_eCDR ("LICHEN-6" to "LICHEN-13", blue), and pairing ("LICHEN-14" to "LICHEN-23", light blue). For pairing_CDRs LICHEN was conditioned to all light therapeutic CDRs, for pairing_eCDR LICHEN was conditioned to essential light CDRs only (CDRL3 for adalimumab, CDRL1 and CDRL3 for pembrolizumab). LICHEN was restricted to make IGKV1, IGKV2, and IGKV3 light sequences and diverse sequences were tested. Binding was tested to the targets of adalimumab (TNF-α) and pembrolizumab (PD-1) ("Target", pink and orange) and the unrelated non-human protein RBD was uses as a control ("Control", light pink and light orange). Phosphate buffered saline ("PBS", black) was used as secondary control. Variants of the pairing use case (LICHEN-14 to LICHEN-23) are not expected to bind to the target as these light sequences are generated without conditioning on any of the light chain CDRs.

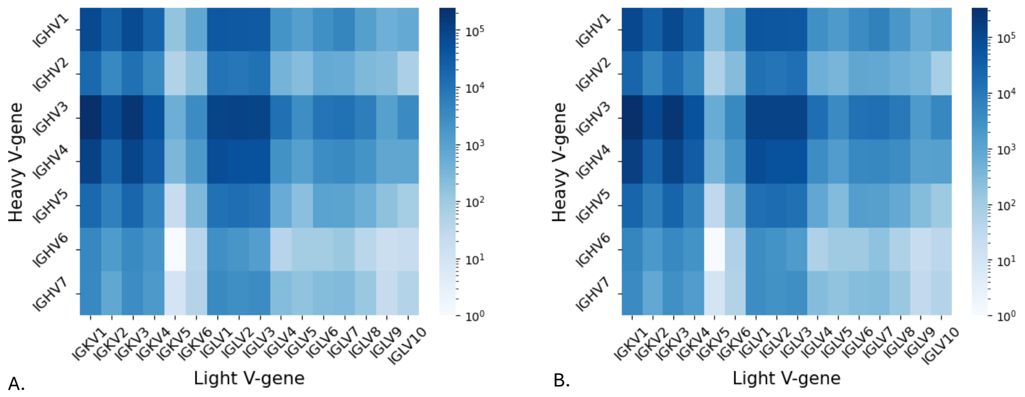

Figure S10. The distribution of V-gene of the heavy and light paired sequences stored in OAS[14] after filtering in November 2023 (A) and June 2025 (B). Similar gene distributions are observed for the 1.8M paired sequences as for the 2.5M paired sequences.

References

1. Dunbar, J. & Deane, C. M. Anarci: antigen receptor numbering and receptor classification. *Bioinformatics* **32**, 298–300 (2016).

2. Greenshields-Watson, A. *et al.* Anarcii: A generalised language model for antigen receptor numbering. *bioRxiv* 2025–04 (2025).

3. Marks, C., Hummer, A. M., Chin, M. & Deane, C. M. Humanization of antibodies using a machine learning approach on large-scale repertoire data. *Bioinformatics* **37**, 4041–4047 (2021).

4. Chinery, L., Jeliazkov, J. R. & Deane, C. M. Humatch-fast, gene-specific joint humanisation of antibody heavy and light chains. *MAbs* **16**, 2434121 (2024).

5. Abanades, B. *et al.* Immunebuilder: Deep-learning models for predicting the structures of immune proteins. *Communications Biology* **6**, 575 (2023).

6. Wong, W. K. *et al.* Scalop: sequence-based antibody canonical loop structure annotation. *Bioinformatics* **35**, 1774–1776 (2019).

7. Schrödinger, LLC. The PyMOL molecular graphics system, version 1.8 (2015).

8. Dunbar, J., Fuchs, A., Shi, J. & Deane, C. M. Abangle: characterising the vh–vl orientation in antibodies. *Protein Engineering, Design & Selection* **26**, 611–620 (2013).

9. Olsen, T. H., Abanades, B., Moal, I. H. & Deane, C. M. Ka-search, a method for rapid and exhaustive sequence identity search of known antibodies. *Scientific Reports* **13**, 11612 (2023).

10. Turnbull, O. M., Oglic, D., Croasdale-Wood, R. & Deane, C. M. p-iggen: a paired antibody generative language model. *Bioinformatics* **40**, btae659 (2024).

11. Raybould, M. I. *et al.* Five computational developability guidelines for therapeutic antibody profiling. *Proceedings of the National Academy of Sciences of the United States of America* **116**, 4025–4030 (2019).

12. Raybould, M. I., Turnbull, O. M., Suter, A., Guloglu, B. & Deane, C. M. Contextualising the developability risk of antibodies with lambda light chains using enhanced therapeutic antibody profiling. *Communications Biology* **7**, 62 (2024).

13. Waterhouse, A. M., Procter, J. B., Martin, D. M. A., Clamp, M. & Barton, G. J. Jalview version 2—a multiple sequence alignment editor and analysis workbench. *Bioinformatics* **25**, 1189–1191 (2009).

14. Olsen, T. H., Boyles, F. & Deane, C. M. Observed antibody space: A diverse database of cleaned, annotated, and translated unpaired and paired antibody sequences. *Protein Science* **31**, 141–146 (2022).
