# Supplementary material for: LICHEN enables light-chain immunoglobulin sequence generation conditioned on the heavy chain and experimental needs
Source: Commun Biol. 2026 Feb 21;9:468. doi: 10.1038/s42003-026-09727-3 (PMC13036001; doi:10.1038/s42003-026-09727-3)
Supplement: Supplementary file 2 — Description of Additional Supplementary Files [file 42003_2026_9727_MOESM2_ESM.docx]

**Description of Additional Supplementary File**

File name: Supplementary Data 1
Description: The numerical source data for the graphs in the main manuscript are provided as an Excel in the Supplementary Data.
